# Indications and technical parameters of cone beam computed tomography in paediatric dentistry at Alexandria and Cairo universities: a retrospective study

**DOI:** 10.1007/s40368-025-01062-1

**Published:** 2025-06-08

**Authors:** M. Baraka, H. M. Ghorab, E. Anter, N. M. ElKersh

**Affiliations:** 1 Alexandria University, Alexandria, Egypt; 2 Cairo University, Giza, Egypt

**Keywords:** Cone beam computed tomography, Conventional radiography, Paediatric dentistry, Dental anomalies, Orthodontics, Bone pathology, Impacted teeth, And Endodontics

## Abstract

**Purpose:**

To investigate the utilization of cone beam computed tomography (CBCT) in paediatric dentistry, focusing on the indications, referring departments, age distribution, oral regions examined, and fields of view (FOV) at Alexandria and Cairo universities.

**Methods:**

This retrospective observational study reviewed records from 2018 to 2024 of patients under 19 years who underwent CBCT scans in the radiology departments. Data were analysed by sex (6–12 years and 13–<19 years), imaging area (anterior/posterior, mandible/maxilla), and indications following the 2011 SEDENTEXCT guidelines: dental anomalies, impacted teeth, endodontics, bone pathosis, orthodontics, and others.

**Results:**

Out of 988 patient records with CBCT scans, 260 paediatric cases were analysed. Referrals mainly came from the oral and maxillofacial surgery department (50%) and the paediatric dentistry department (40%). The primary reasons for CBCT scans included impacted teeth (40%), endodontic treatments (19.6%), and orthodontic assessments (15.8%). Significant usage patterns were noted across age groups, with 53.1% of patients aged 6–12 years and 46.9% aged 13–18 years. A diverse range of oral regions was examined, including the anterior mandible (30%), posterior mandible (25%), anterior maxilla (20%), and posterior maxilla (25%).

**Conclusion:**

The findings reveal that CBCT is primarily used for assessing impacted teeth, endodontic treatments, and orthodontic needs in children and adolescents. Patients aged 6–<13 years were more often referred for endodontic issues, while those aged 13–<19 needed imaging for impactions and orthodontics. Differences in FOV among centres indicate varied clinical practices.

**Clinical significance:**

The study underscores the indications of CBCT in paediatric dentistry and the diverse clinical practices at both universities and highlights the need for tailored imaging protocols.

**Supplementary Information:**

The online version contains supplementary material available at 10.1007/s40368-025-01062-1.

## Introduction

Dental imaging is crucial for diagnosing and monitoring oral diseases as well as planning treatments (American Academy of Pediatric Dentistry, 2024). While traditional methods only provide two-dimensional (2D) images, cone beam computed tomography (CBCT) has enabled the acquisition of high-quality three-dimensional (3D) images (axial, sagittal, and coronal) in dentistry (İşman et al. 2017). This technology offers the advantage of visualizing lesions, teeth, bones, and specific areas of the oral cavity from multiple angles (İşman et al. 2017). Additionally, patients receive a lower radiation dose compared to standard computed tomography (Ding and Munro 2013). As a result of these benefits, the use of CBCT in dentistry has significantly increased in recent years (İşman et al. 2017).

CBCT is primarily utilized in implant dentistry and maxillofacial surgeries, but its application has broadened to encompass all areas of dentistry, including paediatric dentistry (Ding and Munro 2013, Hajem et al., 2020). However, exposure to ionizing radiation carries the risk of permanent DNA damage and mutations that can lead to cancer (Petersen et al. 2015). Because children experience faster cell division during growth, they are two to ten times more susceptible to ionizing radiation compared to adults (Valentin 2007 , Walliczek-Dworschak et al. 2017). This increased sensitivity raises concerns about stochastic radiation effects, making the application of CBCT in paediatric dentistry a contentious issue (İşman et al. 2017). Therefore, it is crucial to modify the radiation dose according to the child's age and body weight, minimize unnecessary repeat imaging, and ensure that the procedure is warranted by appropriate clinical indications (Büyük et al. 2018, Valentin 2007 , Scarfe 2012, Walliczek-Dworschak et al. 2017).

In clinical settings, CBCT is invaluable for assessing calcified tissues (Horner 2013, Scarfe and Farman, 2008). It is frequently recommended prior to maxillofacial and dental implant surgeries, as well as for assessing both benign and malignant tumours, cysts, and other bone pathologies (Dula et al., 2014). CBCT is utilized for a variety of purposes, including examining root canal structures, identifying pathologies, detecting root fractures, planning orthodontic treatments, managing dental anomalies, and evaluating the positions of supernumerary and impacted teeth along with their surrounding tissues. Furthermore, it assists in assessing the morphology and pathologies of the temporomandibular joint, analysing paranasal sinuses and airways, and determining cleft boundaries in patients with cleft lip and palate (Scarfe and Farman, 2008, Acar and Kamburoğlu, 2014, Akarslan and Peker 2015, Dobbyn et al. 2013, Nematolahi et al. 2013, Osorio et al. 2008, Patel et al. 2009). In cases of complex dentoalveolar trauma, CBCT provides significant advantages. However, the decision to order a CBCT scan should always be guided by its diagnostic benefits and a thorough evaluation of the individual case (Dobbyn et al. 2013). Additionally, it is crucial to follow the ALARA principle (As Low As Reasonably Achievable) to minimize radiation exposure while ensuring the highest possible diagnostic quality (Dawood et al. 2009).

Given the potential risks associated with radiation exposure in children, it is imperative for clinicians to have a robust understanding of CBCT's applications in paediatric patients. Standardization and categorization of CBCT utilization are crucial for optimizing its use. Current research on the factors influencing CBCT referrals for children is limited, underscoring the need for ongoing quality assurance and systematic monitoring of radiographic practices in university radiology departments (Hajem et al., 2020, Yiğit et al. 2023). Our study aimed to assess the indications for CBCT referrals in children, the referring departments at the Faculty of Dentistry at Alexandria and Cairo universities, the distribution of referrals across different age groups and oral regions, as well as the technical parameters of the scans. The null hypothesis posited that there is no significant difference in the utilization of CBCT in children based on reasons for requests, referring department, age groups, oral regions, and technical parameters at the Faculty of Dentistry, Alexandria, and Cairo Universities.

## Materials and methods

### Study design and sample size

The current study was a retrospective multicentre cross-sectional analysis that encompassed the records of patients under 19 years of age who were referred to the Radiology unit at the Faculty of Dentistry of Alexandria and Cairo universities for a CBCT scan between January 2018 and September 2024. The study was reported according to STROBE checklist and approved by the Research Ethics Committee, Faculty of Dentistry, Alexandria University (IRB # 00010556-IORG 0008839-0103-10/2024).

For sample size calculation purposes, based on findings reported by İşman et al. (2017), comparisons between age groups in terms of indications would require at least 84 study participants per age group (total sample size of *n* = 168) to achieve a statistical power of 80% under and alpha cutoff value for significance of 0.05. Sample size calculations were performed using the R software for statistical computing version 4.2.1.

Patient records were retrospectively evaluated according to referring department, patients’ age and gender, oral region (anterior/posterior and mandible/maxilla), and the reason for request of CBCT scan. All incomplete records without reasons for referral were excluded. All data were anonymized and stored on a password-protected laptop.

The patients were divided into two age groups: 6–12 years (mixed dentition) group and 13–<19 years (permanent dentition) group. There were no children younger than 6 years of age in this study, since no children younger than 6 years of age had CBCT scans.

**Fig. 1:**
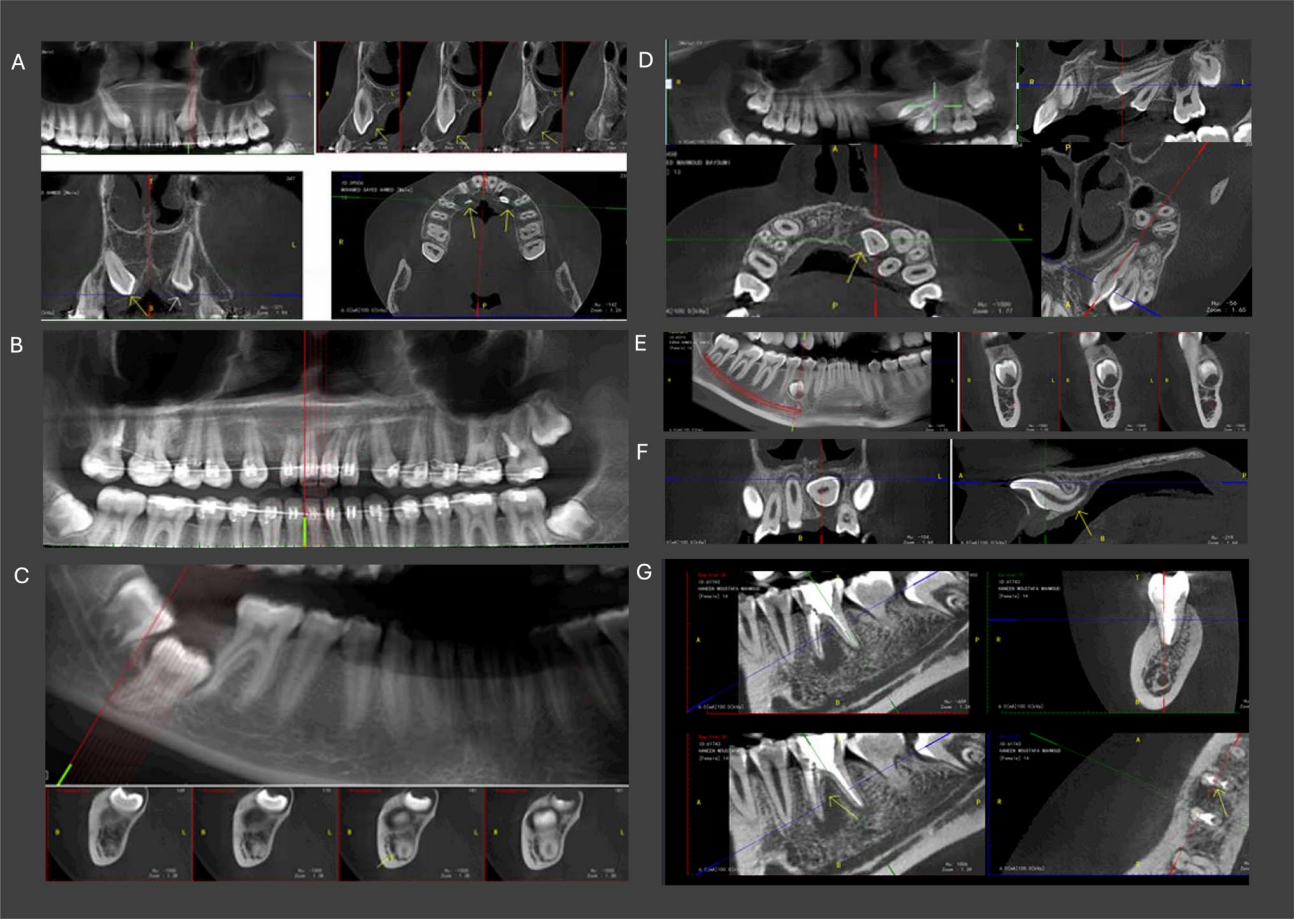
CBCT scans of some patients with different referral reasons. **A** A 13-year-old boy showing impacted canines (impacted teeth group). **B** A 16-year-old girl with orthodontic treatment (orthodontics group). **C** A 17-year-old boy with impacted mandibular right second permanent molar (impacted teeth group). **D** A 13-year-old boy showing impacted left maxillary permanent central incisor with dilacerated root and pericoronal radiolucency (impacted teeth group). **E** A 16-year-old girl showing a supernumerary in the mandibular right posterior region (dental anomalies group). **F** An 8-year-old girl with horizontally impacted maxillary central incisor with dilacerated root (impacted teeth group). **G** A 14-year-old girl showing endodontically treated mandibular right first permanent molar with periapical radiolucency and vertical root fracture of the mesial root (endodontics group)

The reasons for referral were grouped under six headings, based on the 2011 evidence-based set of SEDENTEXCT guidelines, as follows: dental anomalies, impacted teeth, endodontics, orthodontics, bone pathosis, and others. The number (supernumerary teeth, congenital absence of teeth, etc.), size, shape, location anomalies, or structural changes were included in the “Dental Anomaly” category, but “Impacted Teeth” were considered as a separate group. In the “Orthodontic” category, the malpositioned tooth was included. The “Bone Pathosis” category included all bone lesions such as periapical radiolucency, cyst, and tumour. In the “Other” category, postoperative evaluation included implant evaluation, craniofacial anomalies, cleft lip and/or palate, trauma, root fracture, alveolar fracture, and intrusion. Various examples of CBCT scans along with their classification into each group are illustrated in Fig. 1. The CBCT scans were evaluated by two examiners (an oral radiologist and one paediatric dentist), who were calibrated by examining and categorizing ten CBCT scans into one of the six groups, kappa 0.859. In case of disagreement, a consensus was reached by a second paediatric dentist who is the main investigator in the current study.

Besides the total number of referrals in the study age groups, we recorded the following data for participants who underwent CBCT imaging: age at the time of examination (years), gender, requests and indications, technical settings (field of view (FOV) dimensions, mA [tube current] and kV [tube voltage], and exposure time. The data were organized in a Microsoft Office Excel 2016 spreadsheet for further processing.

The CBCT machine used in the Alexandria centre is I-CAT machine (Imaging Science International, 2nd generation, Hatfield, PA, USA) and that used in the Cairo centre is Planmeca (ProMax 3D Mid machine, Helsinky, Finland).

### Outcome measures

- Reasons for referral: dental anomalies, impacted teeth, endodontics, orthodontics, bone pathosis, and others.
- Referring department.
- Patient characteristics.
- Technical parameters of CBCT scans.

### Statistical analysis

Categorical data were shown in counts and frequencies (%) with associations between them being investigated using the Chi square test of independence. A significance level (α) of 0.05 was set to determine statistical significance. The statistical analysis was strictly bound to a pre-specified study protocol and no p value adjustment was required. Data handling and statistical analysis were done using the R programming language for statistical computing version 4.2.1.

## Results

**Fig. 2:**
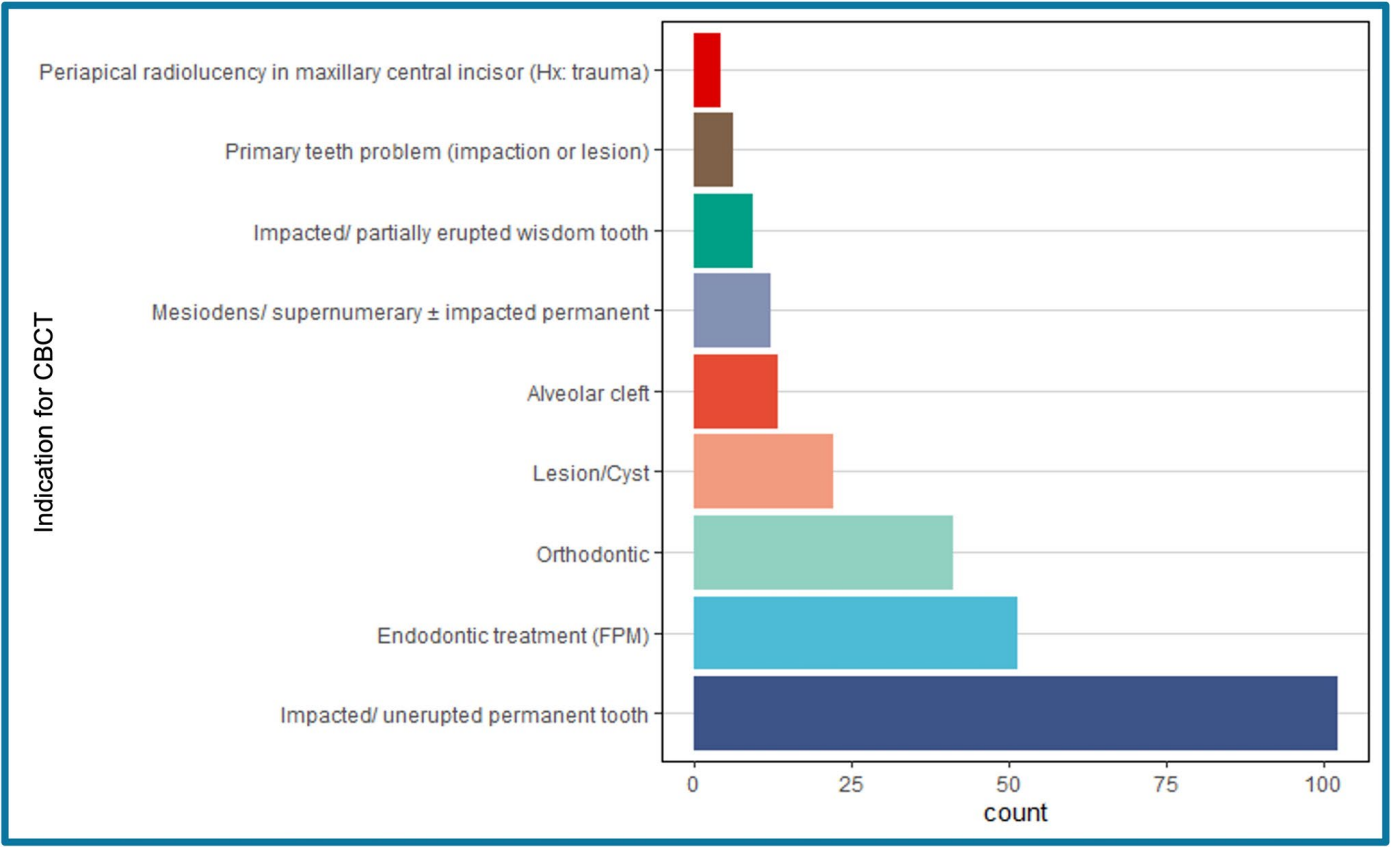
Distribution of CBCT indications among our study sample

Out of 988 patient records with CBCT scans, 260 (26.32%) paediatric cases were analysed with mean age 13.21 years. The records included were 146 from Alexandria University and 114 from Cairo University. The number of patients referred from the paediatric dentistry departments was 104 patients (40%), 130 (50%) from the oral and maxillofacial surgery departments, and 26 (10%) from the orthodontic departments. In terms of technical parameters, all scans conducted at the Alexandria centre used 120/37 Kvp/mA with an exposure time of 26.9 s, whereas scans from Cairo used 120/14 Kvp/mA with a 33 s exposure time. The indications of CBCT scans that were observed in our study before categorization into the six groups are shown in Fig. 2. The main reasons for referring for CBCT scans were impacted or unerupted permanent teeth 102 (39.23%), endodontic indications 51 (19.62%), and orthodontic indications 41 (15.77%). Together, these three reasons accounted for almost 75% of all CBCT scans performed.

Table 1 shows the association between the age groups and the different study parameters. The sample consisted of 147 (56.54%) females, with 43.85% of participants aged between 6 and 12 years, while the remaining 56.15% were aged between 13 and 18 years. There was no significant difference between the gender of study participants and age groups *(p=0.45).* However, cases involving individuals under 13 years of age were significantly more prevalent at the Alexandria centre (82.5%) compared to Cairo (17.5%), *p<0.001*. Regarding referral reasons for CBCT scans, the younger age group showed a significant association with endodontic treatment, mostly of first permanent molar (FPM) (38.6% vs. 4.8%; *p <0.001*). In contrast, indications such as impacted teeth (49.3% vs. 34.2%; *p=0.021*) and orthodontic management (24.7% vs. 4.4%; *p<0.001*) were significantly more common in the older age group. No significant associations were observed for bone pathosis *(p=0.197)*, dental anomalies *(p=0.99)*, or other combined indications *(p=0.37)*. Additionally, full arches were more frequently scanned in the older age group compared to younger participants (23.3% vs. 4.4%; *p<0.001*). The most frequently examined oral region was the maxilla (147 cases, 56.54%). The anterior region was more commonly examined (134 cases, 51.54%) than the posterior region. Various fields of view (FOV) were utilized, with the 8 × 8 FOV being the most prevalent (56.15%), while other FOVs included 16 × 10 (21.15%) and 5 × 5 (15%).

**Table 1:** Association between age groups and study parameters

Term	Overall N (%)	Age group (years)		
		6-12 *N* (%) (*n*=114)	13-18 *N* (%) (*n*=146)	*p*-value
Gender				0.456
Male	113 (43.46)	53 (46.5)	60 (41.1)	
Female	147 (56.54)	61 (53.5)	86 (58.9)	
Centre				< 0.001*
Alexandria	146 (56.15)	94 (82.5)	52 (35.6)	
Cairo	114 (43.85)	20 (17.5)	94 (64.4)	
Indications				
Bone pathosis	26 (10)	15 (13.2)	11 (7.5)	0.197
Dental anomalies	12 (4.62)	5 (4.4)	7 (4.8)	0.99
Endodontic treatment (FPM)	51 (19.62)	44 (38.6)	7 (4.8)	< 0.001*
Impacted teeth	111 (42.69)	39 (34.2)	72 (49.3)	0.021*
Orthodontic	41 (15.77)	5 (4.4)	36 (24.7)	< 0.001*
Others	19 (7.31)	6 (5.3)	13 (8.9)	0.379
Arch				< 0.001*
Full arches	39 (15)	5 (4.4)	34 (23.3)	
Mandibular	74 (28.46)	38 (33.3)	36 (24.7)	
Maxillary	147 (56.54)	71 (62.3)	76 (52.1)	
Location				< 0.001*
Anterior	134 (51.54)	55 (48.2)	79 (54.1)	
Full arches	39 (15)	5 (4.4)	34 (23.3)	
Posterior	87 (33.46)	54 (47.4)	33 (22.6)	
α = 0.05				

*P*-values obtained from Pearson's chi-square test of independence

An overview of the association between the FOV of CBCT scan and the respective centres and indications for the imaging is provided in Table 2. The data indicates statistically significant differences in FOV use between the Alexandria and Cairo centres, with Alexandria predominantly utilizing the FOVs 8 × 8 (63%) and 5 × 5 (26.7%). In contrast, Cairo showed a higher use of the 16 × 10 FOV. The statistical analysis revealed that the choice of FOV is significantly related to both the university *(p<0.001)* and the clinical indication; bone pathosis *(p=0.003)*, endodontic treatment (*p<0.001)*, impacted teeth *(p<0.001)*, orthodontic *(p<0.001)*, and others *(p<0.001).* Endodontic treatments are highly associated with the 5 × 5 FOV, while orthodontic indications were associated with larger FOVs; 29 (52.7%) were 16 × 10 FOV.

**Table 2:** Association between FOV and centre/indication for CBCT.

Term	Overall *N* (%)	FOV						*p*-value
		10*6 *N* (%) (*n* = 1)	14*8 *N* (%) (*n* = 15)	16*10 *N* (%) (*n* = 55)	20*17 *N* (%) (*n* = 4)	5*5 *N* (%) (*n* = 39)	8*8 *N* (%) (*n* = 146)	
Centre								<0.001*
Alexandria	146 (56.15)	0 (0)	15 (100)	0 (0)	0 (0)	39 (100)	92 (63)	
Cairo	114 (43.85)	1 (100)	0 (0)	55 (100)	4 (100)	0 (0)	54 (37)	
Indications								
Bone pathosis	26 (10)	0 (0)	3 (20)	0 (0)	0 (0)	0 (0)	23 (15.8)	0.003*
Dental anomalies	12 (4.62)	0 (0)	0 (0)	4 (7.3)	0 (0)	0 (0)	8 (5.5)	0.552
Endodontic treatment (FPM)	51 (19.62)	0 (0)	0 (0)	1 (1.8)	0 (0)	39 (100)	11 (7.5)	< 0.001*
Impacted teeth	111 (42.69)	1 (100)	3 (20)	14 (25.5)	0 (0)	0 (0)	93 (63.7)	< 0.001*
Orthodontic	41 (15.77)	0 (0)	6 (40)	29 (52.7)	1 (25)	0 (0)	5 (3.4)	< 0.001*
Others:	19 (7.31)	0 (0)	3 (20)	7 (12.7)	3 (75)	0 (0)	6 (4.1)	< 0.001*
α = 0.05								

*P*-values obtained from Pearson's chi-square test of independence

## Discussion

This retrospective study aimed to examine the utilization of CBCT in paediatric dentistry at two major universities in Egypt: Alexandria and Cairo. The findings provide an overview of referral patterns, indications, and technical parameters of CBCT used in children and adolescents.

In the current study, a notable portion of patients, referred for CBCT, were children, with an average age around 13 years, spanning from early childhood to just under 19. This finding aligns with previous studies, such as İşman et al. (2017), which reported a mean age of 13.42 years (range 2–17 years), and Yiğit et al. (2023), with a mean age of 14.32 years (range 6–18 years). The age distribution shows a slight majority in the 13-18 years age group, reflecting a broader range of dental issues that may require advanced imaging as children progress through different stages of dental and craniofacial development. The absence of participants under 6 years of age highlights the principle of minimizing radiation exposure for very young children, who may benefit from alternative imaging methods (Ding and Munro 2013). This observation is consistent with Yiğit et al.’s (2023) study, which noted that most patients were over 13 years old, as children are more susceptible to ionizing radiation than adults. It is believed that clinicians request CBCT more frequently in this context, as children in this age group tend to cooperate better during the procedure. Furthermore, the higher prevalence of impacted teeth— the primary reason for CBCT referrals— in this age group further explains these findings.

Demographic and clinical data analyses indicate significant associations, with the Alexandria centre recording a higher prevalence of scans among younger children (<13 years old), potentially reflecting varying referral patterns or clinical protocols. This younger group showed a strong correlation with endodontic treatments, emphasizing the necessity for accurate imaging with small FOV in managing complex root canal cases in paediatric patients, particularly for permanent first molars with incomplete root formation and deeply decayed molars affected by pulpal involvement and molar incisor hypomineralization. On the other hand, in children aged 13 to under 19 years with permanent dentition, the prevalence of impacted third molars and orthodontic management increased, necessitating larger FOV for comprehensive assessment. This trend underscores the evolving dental needs of children as they grow, transitioning from basic dental health issues to more complex orthodontic and developmental concerns.

The distribution of referrals across different dental specialities highlights the versatile applications of CBCT in paediatric patients. The predominance of referrals from the oral and maxillofacial department aligns with the technology's established role in surgical planning and assessment of complex craniofacial conditions such as alveolar clefts and impacted canines and wisdom teeth (Nematolahi et al. 2013, Patel et al. 2009). The significant contribution from the paediatric dentistry department underscores the growing recognition of CBCT's utility in managing various paediatric dental issues. This comes in agreement with Yiğit et al.’s (2023) study, which reported that the oral and maxillofacial radiology department made the most cbct requests in all the years considered (53.6%), followed by paediatric dentistry department (17.7%). 

The present study, supported by previous data (Yiğit et al. 2023), found that the most common indications for CBCT referrals in the paediatric population were impacted or unerupted permanent teeth, endodontic treatment (primarily of first permanent molars and traumatized upper central incisors), and orthodontic assessments. This aligns with earlier research highlighting CBCT's utility in managing complex dental conditions in children (Akarslan and Peker, 2015, Hajem et al., 2020, İşman et al. 2017, Walliczek-Dworschak et al. 2017, Yiğit et al. 2023, Nahir et al. 2024). The ability of CBCT to visualize these conditions from multiple angles enhances diagnostic accuracy compared to traditional two-dimensional radiographs, which can obscure critical anatomical relationships (Scarfe and Farman, 2008). The high prevalence of scans for impacted teeth, including third molars, underscores CBCT's value in treatment planning for these common developmental issues and its universal relevance in identifying conditions that require precise spatial visualization. Consequently, CBCT is instrumental in assessing complications and planning treatment for impacted teeth (Mossaz et al. 2014).

Studies have indicated that requests for CBCT scans in paediatric patients primarily focus on the maxilla (Helms and Kaplan 1990, Ismayılov and Özgür, 2023, Yiğit et al. 2023). In this analysis, the maxilla was also the most frequently scanned region, particularly the anterior area. This focus aligns with common dental concerns in children, such as impacted or delayed eruption of permanent incisors and dental anomalies like mesiodens (Nematolahi et al. 2013). The significance of the maxilla is underscored by its status as the most common site for supernumerary teeth (Ferrés-Padró et al. 2009, Nematolahi et al. 2013), with the maxillary permanent canine being the second most commonly impacted tooth after the third molar. Additionally, maxillary incisors are the teeth most susceptible to trauma (Mahmoodi et al. 2015), and CBCT imaging proves particularly valuable when superimpositions hinder diagnosis in traumatic cases (Oenning et al., 2018). Conversely, requests related to bone pathologies were predominantly associated with the mandible, aligning with findings from Yiğit et al. (2023).

The study underscores notable differences in technical parameters and preferences for FOV between the Alexandria and Cairo centres. In Alexandria, there was a preference for smaller FOVs, such as 8 × 8 and 5 × 5, particularly for targeted procedures like impacted teeth, dental anomalies, and endodontic purposes, which reflects a strategy aimed at reducing radiation exposure. Only one CBCT scan with a large 16 × 10 FOV was recorded under the endodontics category. Upon reviewing this scan, it was found that the patient had four pulpally involved first permanent molars requiring endodontic treatment. This FOV was intentionally selected to obtain all the required diagnostic information in a single scan, minimizing the number of exposures, even though this came at the expense of reduced resolution. Conversely, the Cairo centre employed larger FOVs (16 × 10) for comprehensive imaging of intricate orthodontic cases. Previous research supports these varying FOV preferences; for instance, Dobbyn et al. (2013) reported that larger scans with a 22 cm FOV were utilized for some orthognathic cases to aid in three-dimensional treatment planning, while a 4 cm FOV was introduced as an alternative to the previously available minimum height of 6 cm in all other orthodontic cases. Among patients under 18 years, a study indicated that 80% of images were captured using a small FOV (5 × 5cm) (Van Acker et al. 2016), while another study reported that a large FOV (20 × 17 cm) was utilized for malocclusion in three-quarters of the cases (İşman et al. 2017). Furthermore, Ismayılov and Özgür (2023) found that small FOVs (≤10 cm) were the most commonly used, whereas large FOVs (23 × 17 cm) were preferred for cases involving malocclusion and dentofacial anomalies. According to the SEDENTEXCT guidelines, large FOVs are particularly advantageous for addressing complex craniofacial deformities in patients aged 16 and older (Horner 2013). However, increasing FOV size significantly raises radiation exposure, especially to sensitive areas like the thyroid and the brain. A paediatric phantom study indicated that the effective dose for a large FOV exceeds 352.5 μSv, compared to below 223 μSv for a small FOV (Marcu et al., 2018). The DIMITRA project recommends using small FOVs (8 × 8 cm) for optimal dose safety and visibility of details (Oenning et al., 2019), although larger FOVs can be necessary for specific indications to balance dose reduction and image quality (Oenning et al., 2018).

Regarding technical settings for CBCT scans implemented in Alexandria and Cairo universities, Alexandria employed a higher mA setting and shorter exposure time, likely to minimize motion artefacts, which is crucial in paediatric imaging. In contrast, Cairo opted for longer exposure times to improve image clarity, weighing the increased radiation risks against diagnostic advantages. However, in both universities, the exposure times were still considered lengthy compared to other settings, as shorter exposure times resulted in low-quality, non-diagnostic CBCT images. Consequently, longer exposure times were chosen for the scans, with proper stabilization of children's heads prioritized to ensure they remain still during imaging. It is noteworthy that the same exposure time was applied to all FOVs, as the dimensions of the FOV or scan volume are mainly influenced by the size and shape of the detector, the beam projection geometry, and the capability to collimate the beam, all of which do not affect the exposure time (Mallya and Lam 2018).

While CBCT technology provides significant diagnostic advantages, the heightened radiosensitivity of children necessitates careful consideration of the risk-benefit ratio for each case. The categorization of referral reasons based on the SEDENTEXCT guidelines reflects an awareness of international best practices (Yiğit et al. 2023), ensuring that CBCT scans are performed only when clinically justified, in line with the ALARA principle (As Low As Reasonably Achievable) or ALADAIP (As Low As Diagnostically Acceptable, Indication oriented, and Patient specific). The International Commission on Radiological Protection (ICRP) emphasizes the need for caution in using dental CBCT for paediatric patients due to their increased radiosensitivity and smaller size. The DIMITRA project has established patient-specific and indication-specific guidelines for the careful use of CBCT in children, advocating for a shift toward an indication-oriented and patient-specific approach to minimize radiation exposure. This underscores the importance of standardization in determining when a CBCT scan is necessary for paediatric patients. On the other hand, the failure to adhere to established low-dose protocols was evident in the parameters of the CBCT scans performed on children at both centres, as efforts were made to improve image quality. Regardless of the CBCT units in use, clinicians should take the initiative to seek optimized alternatives instead of thoughtlessly applying or duplicating the default protocols, particularly when treating children (Yeung et al., 2025).

The increasing trend in CBCT referrals, as highlighted in the present study and supported by Nahir et al., (2024) and Yiğit et al. (2023), necessitates further research into whether the rising use of CBCT may elevate the risk of negative consequences due to increased exposure. Therefore, future studies should prioritize the development of newer technologies or techniques that minimize radiation doses while preserving image quality.

As a retrospective study, this research is limited by the quality and completeness of available records. Future prospective studies could provide more detailed insights into decision-making processes for CBCT referrals and long-term outcomes associated with its use in paediatric dentistry. Additionally, comparing the findings with those from other geographic regions and healthcare systems could offer valuable global perspectives on CBCT utilization in children.

In conclusion, our study revealed that CBCT was appropriately utilized for evaluating impacted teeth, endodontic and orthodontic assessment in paediatric dentistry, at Alexandria and Cairo universities. Most CBCT referrals originated from oral and maxillofacial surgery and paediatric dentistry departments. Notably, 6-<13-year-old patients were more frequently referred for endodontic issues, while 13-<19-year-old patients required imaging for impactions and orthodontic purposes. Additionally, variations in the fields of view used between different centres suggest differing clinical practices. 

## Supplementary Information

Below is the link to the electronic supplementary material.

Supplementary file1 (DOCX 17 KB)

## Data Availability

No datasets were generated or analysed during the current study.
